# Mitochondrial donation and ‘the right to know’

**DOI:** 10.1136/medethics-2016-103587

**Published:** 2016-08-19

**Authors:** Reuven Brandt

**Keywords:** Reproductive Medicine, Philosophical Ethics, Rights

## Abstract

In this paper, I examine two key arguments advanced by the Human Fertilization and Embryology Authority (HFEA) and the Nuffield Council justifying anonymous mitochondrial donation, even though the ‘right to know’ is recognised in standard gamete donation. I argue that the two arguments they offer, what I call the argument from genetic connection and the argument from personal characteristics, are unsuccessful. However, I provide additional reasons for why recognising the right to know in gamete donation but not in mitochondrial donation may be justified. I further argue that the status quo in the UK, which is to not recognise a right to know in mitochondrial donation, is provisionally acceptable.

## Introduction

In 2015, the UK became the first country to approve mitochondrial transfer, which is a clinical strategy aimed at preventing the transmission of pathological mutations in mitochondrial DNA (mtDNA) from affected women to their biological offspring. The two approved transfer techniques, maternal spindle transfer and pronuclear transfer, work by replacing^i^ the mitochondria originating from an affected woman's oocyte with mitochondria from a donor. In the wake of the UK's historic decision, the Institute of Medicine in the USA released a report recommending that studies into mitochondrial transfer be permitted to take place in the USA, perhaps paving the way for wider use of these procedures.^2^

My objective in this paper is not to assess the various arguments made for and against the approval of mitochondrial transfer itself, but rather to examine a particular regulatory question: ought jurisdictions that recognise individuals' right to access identifying information about their gamete donor(s)—what I will call ‘the right to know’—recognise a similar right in the case of mitochondrial donation. This question arises because mitochondria contain their own distinct DNA, and thus mitochondrial transfer results in children created from DNA provided by three individuals.

In the UK, the recommendation of both the Nuffield Council on Bioethics^3^ and the Human Fertilization and Embryology Authority,^4^ which has since been enacted into law, is to not recognise any such right. Two major reasons given for this recommendation are: (1) mtDNA does not encode for phenotypic traits shared between a donor and the resultant child that are constitutive of an individual's unique identity, (***Both the Nuffield Council and the HFEA are quite vague about what kinds of traits they take to be constitutive of personal identity, and there remains much philosophical work to be done in refining this concept. However, as will be discussed later in the paper, some headway is possible with some reasonable speculation.***) and (2) mtDNA donation does not establish a unique genetic connection between mitochondrial donor and resultant child.

In the first section of this paper, I argue that these two reasons are questionable for both philosophical and empirical reasons. However, in the second section, I show that there are other reasons for thinking that recognising the right to know in standard gamete donation cases does not entail recognising the right to know in mitochondrial donation. To be clear, I myself am sceptical that a right to know is even obtained in gamete donation,^ii^ but my argument here is not a general critique of the right to know. Rather, I show that there are substantial disanalogies between mitochondrial donation and gamete donation such that recognising a right to know in the latter does not entail recognising a similar right in the former. In the final section of this paper, I provide an argument for why the status quo in which the right to know is not extended to mitochondrial donation is at least provisionally acceptable, given that there are both relevant symmetries and asymmetries between gamete donation and mitochondrial donation.

Since much of the following discussion will rest on the differences between nuclear DNA (nDNA) and mtDNA, I will briefly outline the biology relevant to the argument that follows.

## Some background biology

nDNA differs from mtDNA in location, inheritance pattern and genomic size. (***This is not an exhaustive list of the differences, but is the difference most relevant to the discussion at hand.***) As the name suggests, nDNA resides in the nucleus of cells. An individual's nDNA is made up of 46 chromosomes, of which half originate from an oocyte and half originate from a sperm cell. By contrast, mtDNA resides in mitochondria, which are a type of cellular organelle. Unlike nDNA, mtDNA is inherited strictly maternally.^iii^ Additionally, when nDNA is (almost) identical^iv^ in all of an individual's somatic cells, individuals can have multiple genetically distinct mtDNA molecules even within the same cell. While this phenomenon, called *heteroplasmy*, was once thought to be rare, more recent studies show that it is in fact ubiquitous.^7–9^

The relative proportions of distinct mtDNA variants are not constant in all cells of the body. By chance, or by some mechanism of mtDNA segregation that is yet to be understood, certain mtDNA variants are present in higher proportion in some cells/tissues than others, including individual oocytes. Consequently, the offspring that result from a woman's oocyte may have different relative proportions of mtDNA variants than the overall relative proportions of the variants found in the woman from whom the oocyte originated.^10^ Additionally, the particular distribution pattern of any pathological mtDNA variants inherited by offspring plays a significant role in the development and severity of any subsequent mitochondrial disease.^9^ ^10^ As a consequence of heteroplasmy, a woman who herself is asymptomatic may have some oocytes containing a high proportion of pathological mtDNA and, thus, might produce some offspring with mitochondrial disease, and others that are symptom-free. Prior to the approval of mitochondrial transfer techniques, women carrying pathological mutations were consigned to either risk having children with mitochondrial disease (***Some examples include Leigh's disease, Leber hereditary optic neuropathy (LHON) and Barth syndrome.***) or forgo having genetically related children altogether. The newly approved techniques permit women at risk of having children with mitochondrial disease to use mtDNA from a healthy ova donor, while still enabling them to pass their own maternal complement of nDNA to their offspring. The end result is a child who is genetically related to his/her intending mother through nDNA, but is also genetically related to his/her mitochondrial donor through mtDNA.

### The argument against ‘the right to know’

Mitochondrial donation results in a child who inherits genetic material from three different people: nDNA through the sperm and ova of two individuals, and mtDNA through the ova of an additional individual. This is a departure from standard forms of donor-assisted reproduction, in which mtDNA is inherited from the same ovum that provided the maternal complement of nDNA. Given that many jurisdictions recognise children's right to know in the standard gamete donation case, a question raised by the newly approved techniques is whether a similar right to know ought to be extended to children created via the use of donor mitochondria.

The recommendation from the Nuffield Council on Bioethics is that no such right ought to be recognised. A key reason for this recommendation in the claim that mtDNA is importantly different from nDNA with respect to the kind of phenotypic traits that it influences and also to the kind of connection it establishes between offspring and donor. They state,

[I]t is discordant with current cultural conventions generated around (nuclear) genetic parenthood, that (as far as we are aware) mitochondrial genes convey to the resulting child no physical resemblances or other traits of personal characteristics of the donor, beyond that of health or ill-health. It is also discordant that mitochondrial genetic contributions (usually) create no identifying or distinguishable link between the resulting child, and their mother (or donor, in the case of cell reconstruction) … Taken alone, mitochondrial genes do not uniquely link the resulting child to their donor in the same way that a donation of nuclear genes would do, and may not give the child a mitochondrial genome distinguishable from those carried by several other close relatives on their donor's side. (4.113)^3^

At least one of these factors, the kind of phenotypic characteristics mtDNA is thought to influence, also features in public opinion about the right to know in mitochondrial donation. In the HFEA's public consultations leading up to their recommendation, they found that, ‘Those that saw [mitochondrial donation] as different from gamete donation often said “it won't determine the characteristics of individuals; it will simply prevent them from inheriting a genetic disease.”’ (6.31).^4^ Similarly, in their summary of public opinion on the matter they report that,

As mitochondria are thought not to be responsible for a person's characteristics (beyond their health), information about a mitochondria donor's personal details and identity should only be disclosed on a basis of mutual consent through a system without a statutory standing. (6.57)^4^

It is worth noting that there is a subtle difference between the argument made by the Nuffield Council and the opinion expressed in the HFEA report. Whereas the Nuffield Council's argument rests on whether *resemblances* between a donor and the resultant child are conveyed via mtDNA, the opinion expressed in the HFEA report seems to rest on the more general claim about the effect mtDNA donation has on personal characteristics, without any mention of resemblance to the donor. However, it seems implausible that mere effect on personal characteristics could ground a right to know. Consider that the actions of the technicians involved in mitochondrial transfer procedures will affect the personal characteristics of the resulting children because they influence which particular gametes will come together to form zygotes. However, it seems unreasonable that children created using donor mitochondria (or donor gametes for that matter!) ought to have access to identifying information about the members of their medical teams, even if these individuals do influence their personal characteristics. Despite any reference to resemblances in their statement, a more charitable reading of the HFEA's position is that since mtDNA is not taken to influence personal characteristics, mtDNA donation cannot establish genetically based resemblances in personal characteristics between donors and the children they help create.

We are thus presented with two arguments for why we should not extend the right to know to mitochondrial donation cases. The first, which I will call the ‘the argument from personal characteristics’, can be summarised as follows:

1. Transfers of genetic material from the donor to the resultant child that result in resemblances in personal characteristics between the two can ground the right to know.
2. mtDNA donation does not give rise to resemblances in personal characteristics between the donor and the resultant child.
3. Therefore, mtDNA donation does not give rise to a right to know on the basis of resemblances.

The second, which I will call ‘the argument from genetic connection’ can be summarised as follows:

1. A unique genetic connection between progenitor and offspring can ground the right to know.
2. No unique genetic connection is established between mtDNA donor and resultant offspring.
3. Therefore, mtDNA donation does not ground the right to know on the basis of a unique genetic connection.

#### Assessing the argument from personal characteristics

First, let us consider the argument from personal characteristics. While the HFEA and the Nuffield Council consider resemblances in personal characteristics to play an important role in grounding the right to know, there is room for disagreement about whether this is indeed the case. For example, Françoise Baylis argues that narrative history is important to individuals' sense of identity in a way that transcends appearances and character traits, and that consequently it is proper to think of children created using donor mitochondria as having three biological parents.^11^ Though Baylis does not explicitly state what implications this account of identity have for the right to know, it is not a stretch to imagine a defender of this position thinking that knowing the identity of one's biological parents is important for an accurate narrative history. On this view then, resemblances between the donor and the child are not *necessary* for a right to know to arise. Conversely, authors have also argued that resemblances are not *sufficient* for the right to know. Consider that in her defence of anonymous gamete donation and closed adoption, Sally Haslanger convincingly argues that the importance placed on the purported resemblances between children and their biological relations is greatly overstated.^12^

Despite the reservations we may have about the importance of resemblances in personal characteristics for the right to know, it is still useful to examine what follows if we do grant this view for the sake of argument. This is in part because, as revealed by the consultations undertaken by the HFEA and the Nuffield committee, many people in fact take this first premise of the argument to be true.

Turning then to the second premise of the argument, what is at issue is whether mtDNA donation results in similarities in personal characteristics between the donor and the resultant offspring. While both the Nuffield Council and the HFEA accept that donor mtDNA is unlikely to affect the personal characteristics of the children born following mitochondrial transfer, neither offer a definition of ‘personal characteristics’. (***The Nuffield report offers sex as an example of a personal characteristic, but does not attempt to define the category in any great detail.***) Their omission makes assessing the standing of this empirical claim difficult and forces us to speculate about what kinds of characteristics the HFEA and the Nuffield Council had in mind. I suggest that a reasonable way to proceed is to look at the kinds of characteristics that are appealed to when defending the view that there is value in having contact with one's genetic relations on the grounds of resemblances. Perhaps, the most well-known advocate of such a view is David Velleman, and on his account resemblance in physical appearance and personality traits play a prominent role.^13^ We can thus reasonably speculate that genetic sequences that influence personality and physical appearance would likely count as influencing personal characteristics, while genetic sequences governing deep biological processes that bring about no normally observable difference between individuals would not. For instance, a series of genes that influence one's behavioural response to stress may count as affecting personal characteristics, while genes that cause pragmatically insignificant changes to rate of hair growth likely would not.

As noted by Bredenoord *et al*,^14^ contrary to the views expressed by the HFEA, it seems plausible, if not highly likely, that mitochondrial transfer does affect the personal characteristics of the resultant offspring. Someone who does not suffer from a debilitating disease from birth is likely to have a very different personality than someone who is free of such disease. Additionally, as Bredenoord *et al* also state, there is evidence suggesting that mitochondrial disease affects cognition and behaviour. (***More on this below. ***) However, as stated previously, a mere effect on personal characteristics is not sufficient to ground a right to know; if it did it would lead to an implausible expansion of individuals whose personal information would have to be made available. The more plausible view is that the resemblances between the donor and the resultant child are what give rise to a right to know.

It is possible that although mtDNA affects personal characteristics as suggested by Bredenoord *et al*, when diseased mitochondria are replaced with those from a donor, the personal characteristics that then arise are encoded by nDNA. What then might result are resemblances between the child and the nDNA provider, but not the mitochondrial donor. Consider the following hypothetical example. Imagine that some mtDNA sequence results in children born without any hair by impeding the expression of genes involved in the production of follicles. Once this mtDNA is replaced with a donor's, the follicles develop according to gene sequences located in nDNA. The result is a child whose hair characteristics resemble the individuals who provided the nDNA and not the mtDNA, though the donated mtDNA did have an effect on the expression of the characteristic. By contrast, we could also imagine a mtDNA sequence that directly encodes a specific trait—say hair colour—and thus results in a shared characteristic with a mtDNA donor. We can therefore distinguish between mtDNA donation that only affects personal characteristics, and mtDNA donation that also results in resemblances in personal characteristics with mtDNA donors. Only the latter would give rise to a right to know.

What matters then is whether mtDNA transmits donors' characteristics to the resultant children. Recall that the Nuffield Council and the HFEA accept the claim (at least provisionally) that mtDNA has no meaningful phenotypic significance at all beyond conferring health or ill health. A plausible reading of this claim is that any phenotypic differences that might arise from mitochondrial donation are due to the restoration of traits that were inhibited by pathological mtDNA, as in the hypothetical hair growth example. However, the empirical evidence is not so clear. There is a growing body of research associating specific mtDNA variants with particular phenotypic traits including personality,^15^ ^16^ psychological disorder^17–19^ and propensity for developing degenerative neurological diseases.^20^ Of these examples, the link between mtDNA and non-pathological behavioural traits, such as extroversion, is the weakest, and so one may argue that these examples at most suggest that certain pathologies, such as bipolar disorder and certain forms of dementia, are in fact hitherto unrecognised mitochondrial diseases. Consequently, the view that mtDNA confers nothing but either health or ill health might still remain unthreatened. Despite this possible interpretation of the evidence, there is good reason to question the conclusion that mtDNA does not impart resemblances to offspring.

Many psychiatric disorders exist on a continuum with behavioural traits that are within the non-pathological range of behavioural norms. Consequently, it is well within the realm of possibility that individuals who inherit milder forms of mutations similar to those suspected to be involved in psychiatric disorders might inherit behavioural characteristics in the non-pathological range. The possibility of non-pathological behavioural effects is made even more plausible in light of a particular feature of mtDNA inheritance previously mentioned—heteroplasmy. As noted previously, individuals can inherit multiple different mitochondrial genomes and thus can have differing degrees of penetrance of mutated mtDNA in their tissues. It thus may well be the case that some elements of personality are inherited via variants in mtDNA even in the absence of disorder.

This argument does not definitively establish that particular mtDNA variants encode specific personality traits in individuals free of mitochondrial disease, but it does show that the possibility that mtDNA has such effects is not out of keeping with current research into the role of mtDNA. Furthermore, it is worth noting that the link between nDNA and personality is itself hotly contested—a recent article argues that a meta-analysis of research examining the genetic inheritability of personality suggests a null hypothesis. The authors of this study even state that, ‘the search for genetic mechanisms of human personality, in our view, will never bear fruit’.^21^ There are of course others who disagree.^22^ The upshot here is that unless the view is that physical resemblance alone (for which there *is* unequivocal evidence of genetic inheritance) is sufficient to ground a right to know, it seems hard to firmly ground the ‘discordance’ between the phenotypic importance of nDNA and mtDNA on the available empirical evidence; in both cases, there is much uncertainty. Given the absence of a detailed understanding of the genetic basis of personality in general, and the evidence that mtDNA may indeed play a role, it is overly hasty to justify policy on the grounds that mtDNA has not yet conclusively been shown to play a role in the transmission of personality traits.

Taking a definitive policy stance in the face of empirical uncertainty could risk causing problems down the line, if future research establishes that mtDNA plays a role in the transmission of personal characteristics. Either children born using donated mtDNA would have to continue to be denied a right to know in spite of such findings, or a retroactive change in policy would have to impinge on the privacy interests of individuals who had donated mtDNA with the understanding that their identity would be protected. The latter is not a mere theoretical possibility, as evidenced by recent controversies in Australia^23^ and British Columbia.^24^ Neither denying children their rights nor retroactively changing the law are appealing outcomes. Given the empirical uncertainties, a less definitive policy is likely more appropriate. Instead of outright declaring that no right to know arises in mitochondria donation, policy could reflect the current empirical uncertainty by explicitly leaving open the possibility of revisiting the question. For instance, potential donors could be informed that though the current state of evidence does not establish a right to know, if this were to change then mtDNA recipients would be granted access to identifying information about their donors. This would provide individuals strongly opposed to having their identity revealed with forewarning about possible retroactive changes in policy, while at the same time building in regulatory flexibility that is appropriate, given the current state of evidence.

#### Assessing the argument from genetic connection

Consider now the second argument, that there is no right to know in the absence of a ‘unique genetic connection’ to a donor. Here again, the Nuffield Council declines to define their terms, so some speculation about what they mean is required. Two possibilities seem to naturally present themselves. The first is a pragmatic criterion: a donor and the child share a unique genetic connection iff it is possible to uniquely identify the donor as the child's progenitor on the basis of genetic analysis alone. The second is an ontological criterion: a donor and the child share a unique genetic connection iff it would have been impossible (or highly unlikely) for the child to have the same genetic makeup but a different donor. The argument from genetic connection can therefore be presented in the following manner:

Since mitochondria do not undergo recombination, and mitochondrial inheritance is strictly maternal, any one of the donor's close female family members could have served as a substitute mitochondrial donor without affecting the mitochondrial genome inherited by the resultant child. Consequently, on both the pragmatic and ontological reading of the criterion, mitochondrial donation does not result in a unique genetic connection between donor and child. Therefore, no right to know arises.

However, on closer inspection the argument is not as plausible as it may initially seem.

First, the pragmatic criterion fails to capture our intuitions about the standing of the right to know in gamete donation cases involving monozygotic siblings. Consider a child created using the sperm of a man who was a member of a set of monozygotic triplets. Genetic testing alone would be unable to determine which member of the three siblings was in fact his/her progenitor, and so on the pragmatic reading of the criterion there would be no unique genetic connection between the child and the man who in fact donated sperm. However, it seems problematic to deny children created in such circumstances the right to know while extending this right to other donor-conceived children.

The ontological reading of the uniqueness criterion better captures our intuitions in the monozygotic sibling example because it provides justification for treating as important the link between the monozygotic sibling who actually donates gametes and the child that results. This is because the individual who in fact donates determines the exact combination of nuclear genes that are passed on to the resultant offspring. Consider that if one of the siblings would have stood in place of the actual donor, the resultant child would have, in overwhelming likelihood, inherited a different complement of paternal nDNA. Consequently, there is a kind of non-fungible link between the particular donor and the child that is created.

Someone may argue that the ontological reading demonstrates that mtDNA does not create a unique genetic link, since there is no recombination of mtDNA and so no ‘shuffling’ of the genes that get passed on to subsequent generations. We might then presume, as does the Nuffield Council, that there would be no variation in the mtDNA inherited by a child even if one of the donor's close female relatives would have donated instead. This would be unlike the sperm donation case, where the identity of the individual who in fact donates plays a role in determining the precise nDNA that is inherited by the resulting offspring.

However, this conclusion does not take into consideration the phenomena of heteroplasmy described earlier in the paper. Since different tissues possess different relative amounts of mtDNA variants, including variations in mtDNA ratios in ova, the precise makeup of mtDNA variants passed on will depend on the specific ovum used in mitochondrial donation. Consequently, the particular individual who in fact donates mitochondria does play a role in determining the specific mtDNA inheritance of the resulting child, and thus on the ontic criterion a ‘unique genetic connection’ could arise.

### A different argument against the right to know

Up to this point, I have argued that the reasons offered by the HFEA and the Nuffield Council for dismissing the right to know in mitochondrial donation cases do not succeed. However, that their justifications are inadequate does not imply that their conclusion is false. In the remainder of this paper, I sketch some reasons why I think the right to know as defended in gamete donation need not be extended to mitochondrial donation, despite the considerations raised in the previous section. In making this argument, I will be following in the path of Kimberly Leighton, who argues that a right to know in gamete donation cannot be defended via an argument from analogy to the right to know recognised in adoption.^25^ Though highlighting disanalogies between gamete donation and mitochondrial donation does not conclusively establish that there are no plausible grounds for extending the right to know to mitochondrial donation, it does shift the burden of proof to those who favour a right to know in mitochondrial donation cases, who must then provide positive arguments for why we should accept such a right. This is so especially because the right to know is generally not defended on the basis of genetic connection and phenotypic resemblance alone.^v^

To begin, consider the differences in family composition that are likely to result from the mtDNA donation and gamete donation even if we presume that mtDNA is of similar importance to nDNA. In standard cases of gamete donation without identity disclosure, one of four possibilities is likely to be the intended result: (1) a child who is to be raised by a single parent who is his/her genetic parent, and thus the child would be aware that he/she has another genetic parent that he/she does not know; (2) a child who is to be raised by two or more parents and is led to believe that two are his/her genetic parents though only one is; (3) a child who is to be raised by two or more parents and is informed that only one parent is his/her genetic parent, and like in the first case would be aware that he/she has not met one of his/her genetic parents, and (4) a child who is not parented by any genetic parents but believes that he/she is parented by two genetic parents. Thus, in the case of gamete donation without identity disclosure, the child will at most have intimate contact with one person whom he/she both believes is his/her genetic parent and is in fact his/her genetic parent. By contrast, in cases of mitochondrial donation without identity disclosure, only one possibility is likely to be the intended outcome, one in which a child has intimate contact with two individuals whom he/she both believes to be his/her genetic progenitors and who are in fact his/her genetic progenitors. This difference has consequences for any presumption of symmetry between gamete donation and mitochondrial donation when it comes to non-disclosure.

For instance, consider Velleman's claim that contact with one's genetic relations is important because crucial self-knowledge is gained through contact with those whom we resemble.^13^ On his view, disclosure of identifying information would be important as a (perhaps distant) ‘second best’ to being raised by one's genetic parents because it at least offers an avenue for children to attempt to initiate contact with their progenitors later in life. Even if we grant Velleman his controversial view about self-knowledge and genetic ties, there is still reason to the think that the case for disclosure is much weaker in the case of mitochondrial donation. This is because even though mtDNA may result in resemblances that are important to self-knowledge, the majority of inherited traits are likely transmitted via nDNA. Consequently, a child created using donor mtDNA still develops intimate relationships with the genetic progenitors responsible for the vast majority of their inherited traits. That is not to suggest that traits conferred via mtDNA are insignificant, but only that individuals who are raised by both their nDNA progenitors but not their mtDNA donor will be lacking less of the self-knowledge that comes from contact with close genetic relations than those raised by only one nDNA progenitor. Given that there is less of a loss of the crucial good, self-knowledge, it may well be the case that other interests, such as those of the intending parents and mtDNA donors themselves, supersede the interests children have in having access to identifying information about their mtDNA donors.

Additionally, if we think that any harms that might arise from lack of knowledge about one's genetic progenitors are socially constructed rather than grounded in biological facts,^12^ there are also reasons to think that there will be substantial asymmetries between gamete donation and mitochondrial donation. Consider that up until recently, it has not been possible to separate the transmission of the maternal complement of nDNA from the transmission of mtDNA, nor has any importance been traditionally placed on mitochondrial inheritance. By contrast, in the West rearing arrangements that involved individuals other than a gestational mother and the child's genetic father carried with them certain stigmas for both the parents (eg, shame and condemnation associated with reproduction outside of marriage) and child (eg, illegitimacy, and its legal and social consequences).^25^ It is therefore unclear whether the socially constructed desire ‘to know’ and related frustration and distress some children created via donor gametes experience will equally arise in children created via donor mitochondria. We cannot simply assume that the experience of children created via anonymous mitochondria donation will parallel that of children created as a product of anonymous gamete donation.^vi^ Consequently, arguments against anonymous gamete donation that are based on the emotional distress reported by children who have a deep desire ‘to know’ cannot be extended a priori to anonymous mitochondrial donation.

A further argument offered in defence of the right to know arises from individuals' health interests. Here, the claim is that in many cases medical histories are used for determining the appropriateness of certain kinds of medical tests, or for diagnosing illnesses that might have a congenital element. In gamete provision cases, one worry is that individuals might seek invasive medical tests for diseases they are unlikely to have because they incorrectly assess their risk of developing diseases by including in their estimation the medical history of a social parent who they mistake for also being a genetic progenitor. Such individuals might also alter behaviour or suffer psychological strain as a consequence of overestimating their risk of developing certain diseases. An additional but related worry is that in the absence of medical information from their genetic progenitors, children born through the use of donor gametes are denied access to information that might be valuable for avoiding or diminishing the severity of a disease for which they are at a heightened risk of developing. Though much of this medical information could be provided via anonymity-preserving mechanisms (eg, medical profiles provided by clinics),^27^ some worry that in order to facilitate getting up-to-date information, especially later in life, offspring ought to have identifying information about their gamete donors.^28^

It is worth noting that some have argued that the value of medical histories to the health interests of children is somewhat exaggerated.^5^ However, even if we take medical interests to justify (even in part) the right to know in the case of gamete donation, there are some reasons to think this justification does not equally apply in the case of mitochondrial donation. First, in mitochondrial donation, the absence of a right to know would only compromise information about diseases in which the mitochondrial genome is implicated, which is a much smaller class of potential diseases, and a class of diseases for which potential donors are screened. Second, because mitochondrial donation does not always remove all pathological mtDNA, follow-up and education about mitochondrial disease may still be recommended for individuals created using mitochondrial transfer. Additionally, female children created using mitochondrial donation who are themselves asymptomatic but nevertheless inherit small amounts of mutated mitochondria might still risk transmitting mitochondrial disease to their offspring, if some ova develop with a large proportion of mutated mtDNA.^5^ Consequently, some degree of precaution prior to future reproductive decisions may be warranted. These last two reasons suggest that children created from donor mitochondria likely benefit to a certain extent from the medical information from their mother whose mtDNA was replaced. No such benefit arises in the case of gamete donation.

These disanalogies demonstrate that additional philosophical work must be done in order to claim that children created using donor mitochondria have the same right to know as individuals created using donor gametes.

## Policy conclusions

So far I have shown that neither of the arguments offered by the HFEA and the Nuffield Council succeed in establishing that the right to know should not be extended to children created using donor mtDNA. This is because (1) empirical evidence suggests that mtDNA might transmit personal characteristics in a manner that results in resemblances between donor and resultant child and (b) mtDNA in fact does create a unique genetic connection with offspring on at least one plausible account. However, I have also argued that there are substantial asymmetries between standard gamete donation and mtDNA donation that provide different reasons for questioning extending the right to know to mitochondrial donation. My analysis therefore suggests that there are both important similarities, namely possible transmission of personal characteristics and the presence of a unique genetic connection, and important differences between gamete donation and mitochondria donation. There is much room for disagreement about how much weight ought to be placed on the similarities and differences I have highlighted, and thus the preceding analysis does not fully resolve the question of whether we ought to recognise a legal right to know in mitochondrial donation.

A complete analysis of whether consistency demands that we extend the legal right to know to mitochondrial donation would require both a greater understanding of the phenotypic significance of mtDNA than that currently exists, and a more in-depth examination of the asymmetries highlighted in the previous section than the scope of this paper permits. However, I will nevertheless offer a justification for provisionally accepting the approach taken by the UK, which is not to extend the legal right to know to mitochondrial donation.

Currently, mitochondrial transfer is the only option available for women who have a deep desire to have genetic offspring but are at risk of transmitting mitochondrial disease to subsequent generations.^vii^ Policy that guarantees the right to know may dissuade individuals from taking advantage of this treatment option and may result in individuals risking having children with severe mitochondrial disease in order to have genetically related children. Furthermore, not legally recognising the right to know still allows individuals who wish to enter into open donor arrangements to do so. Consequently, the status quo is likely more attractive to individuals considering mitochondrial donation than the alternative. Given that mitochondrial diseases can be quite severe and transmissible to subsequent generations (in the case of female offspring), the potential harm caused by policies that discourage individuals committed to having genetically related children from pursing mitochondrial transfer is likely to be quite high. Though perhaps biased by my views on the right to know in general, I am inclined to think that policy that results in more children being created with mitochondrial disease is likely to be more harmful overall than policy that results in children who are not given access to information about their mitochondrial donor—especially given the arguments advanced in the preceding section outlining why these harms will likely be less severe than any similar harms experienced in the standard gamete donation case. Thus, while I think that at best the current state of evidence only justifies a less definite policy of the kind briefly outlined earlier in the paper, for pragmatic reasons the status quo is defensible for the time being.

This argument does not commit me to a particular stance on the relevance of the non-identity problem to children created using donor mitochondria because it does not rest on a claim about whether a particular child is made better or worse off.^viii^ Rather, the suggestion is that given the current ethical and empirical uncertainties, policy makers should opt for the regulations that are likely to result in the lowest amount of overall harm in society.^ix^ To be clear, I am not claiming that, in general, we ought to settle questions about the right to know on utilitarian grounds alone. Rather, my thought is that in the absence of sufficient information to fully settle whether appeals to parity demand that we extend the right to know to mitochondrial donation, and given the need to have some policy in place, a harm-based approach seems to be an appropriate way to proceed. Of course, this may mean reversing the policy at a later date, putting us in the situation discussed previously where we will either have to retroactively change the policy or deny some children the right to know their mitochondrial donor. Unfortunately, this is likely the best we can do given our current state of knowledge.

## Footnotes

i The procedure is not perfect, and some of the mitochondria from the intending mother may be passed on to the subsequent offspring.^1^

ii Other authors have advanced convincing arguments for why we ought to be sceptical of the right to know in gamete donation.^5^

iii There is some debate about whether mitochondrial inheritance is strictly maternal, as in rare cases mitochondria found in the agella of sperm cells can become incorporated into the zygote.^6^ However, at the moment the orthodox view is that mitochondrial inheritance is maternal.^7^

iv Spontaneous mutations will result in small differences, and in rare cases individuals who are natural chimeras may have radically different genomes in different tissues.

v Lucy Firth provides a good overview of the debate.^26^

vi Both the HFEA and Nuffield reports state that mitochondrial donation ought to be seen as different in kind from standard gamete donation when it comes to disclosure of information.^3^ ^4^

vii However, there are some exciting new treatment developments on the horizon.^29^

viii Wilkinson *et al*. offer an analysis of the particular consequences the non-identity problem has that are specific to mitochondrial donation.^30^

ix This suggestion is somewhat similar to Derek Parfit’s ‘Same Number Quality Claim’. For a discussion of the application of this principle to assisted reproduction, see Wilkinson.^31^
